# Sexual dysfunction outcomes and reporting disparities after multimodal treatment for rectal cancer: A systematic review and narrative synthesis

**DOI:** 10.1111/codi.70599

**Published:** 2026-08-28

**Authors:** Vignesh Lakshmanan, Jennifer Waterman, Alun Meggy, Jared Torkington, Julie A. Cornish

**Affiliations:** ^1^ Department of General Surgery University Hospital of Wales, Cardiff and Vale University Health Board Cardiff UK; ^2^ Clinical Research Facility University Hospital of Wales, Cardiff and Vale University Health Board Cardiff UK; ^3^ Cardiff University Cardiff UK

**Keywords:** female sexual function, patient‐reported outcome measures, rectal cancer, reporting disparities, sexual dysfunction, survivorship

## Abstract

**Background:**

Sexual dysfunction is a recognised adverse outcome after rectal cancer treatments, yet reporting is predominantly male‐focused, with female sexual dysfunction less consistently assessed or reported. This systematic review aimed to assess sexual health domains studied, examine sex‐based reporting disparities, and identify limitations in the current evidence.

**Methods:**

A systematic review and narrative synthesis were conducted in accordance with PRISMA and registered with PROSPERO (CRD420251178502). PubMed/MEDLINE, EMBASE, Scopus, CINAHL Plus, PsycINFO, and Cochrane Library were searched from March 1995 to March 2025, with an updated search to March 2026. Eligible studies included adults treated for rectal cancer in whom sexual function outcomes were assessed using International Index of Erectile Function (IIEF) and/or Female Sexual Function Index (FSFI), with additional related patient‐reported outcome measures (PROMs) accepted where relevant. Risk of bias was assessed using RoB 2 and MINORS.

**Results:**

Twenty‐five studies involving 4,071 patients were included. Participants were predominantly male (65.8%), while women comprised 34.2%. PROM reporting was inconsistent; only eight studies reported both total and domain‐level results. Sexual outcomes were most consistently reported for men, particularly erectile and ejaculatory function, whereas female outcomes were often reduced to overall scores. Five studies restricted sexual outcome assessment and analysis to men. Only six reported response rates, and missing PROM data handling was not described. Key contextual variables, including sexual activity, sexual orientation, sexual practices, and menopausal status, were rarely captured.

**Conclusions:**

Sexual function outcomes after rectal cancer treatment are inconsistently reported, with low certainty. Female sexual dysfunction remains under‐represented. Standardised, inclusive sex‐specific reporting and development of a core outcome set are needed.

**What does this paper add to the literature?**

This systematic review highlights important sex‐based reporting disparities in rectal cancer survivorship. It shows that women remain underrepresented, female sexual dysfunction and associated clinical factors are inconsistently assessed, and current outcome reporting lacks standardisation which limits equitable understanding of survivorship after rectal cancer treatment.

## INTRODUCTION

Colorectal cancer is the fourth most common cancer in the United Kingdom, accounting for approximately 11%, and rectal cancer represents around one‐quarter of diagnoses [1]. Advances in multimodal, curative‐intent treatments have improved oncological outcomes and survival [2, 3, 4]. However, treatment‐related morbidity varies, and sexual dysfunction is a well‐recognised adverse outcome that can substantially affect quality of life and psychosocial wellbeing [5, 6, 7, 8].

As survival has improved, attention has increasingly turned to long‐term quality of life (QoL) outcomes, including genitourinary and sexual dysfunction following surgery, radiotherapy, and other treatments for rectal cancer [9, 10]. Existing literature has predominantly focused on male sexual outcomes (mainly erectile and ejaculatory dysfunction), while female sexual outcomes (including dyspareunia, vaginal dryness and reduced desire) are less consistently studied [11, 12, 13]. Lee et al. [14] highlighted sex‐based disparities in rectal cancer surgery and noted that much of the sexual dysfunction literature remains male‐centred.

Female sexual dysfunction has often been relatively overlooked and under‐reported, with some studies suggesting that women's reluctance to discuss sexual issues may contribute to this underrepresentation [8, 15, 16]. Sexual wellbeing is often a multifactorial late effect of cancer treatment; however, it may be overlooked in routine follow‐up, and patients may be reluctant to raise concerns. A report from the cancer charity, Macmillan, highlights that sexual function‐related problems can be forgotten or not discussed despite their impact on quality of life [17]. International survivorship guidance, from the National Comprehensive Cancer Network (NCCN), and the American Society of Clinical Oncology (ASCO) recommendations emphasise routine, multidomain assessment of sexual health, including psychosocial context and distress as part of comprehensive survivorship care [18, 19].

Despite this, evidence of sexual health outcomes in rectal cancer management remains sparse and is often obscured by the lack of inclusive collection of data such as sexual orientation, practices, preferences, and menopause status within cancer services [20, 21, 22]. There is increasing need for studies that comprehensively assess sexual function, intimacy, sexual orientation, and sexual practices [23, 24].

This systematic review therefore aimed to synthesise the available evidence on sexual dysfunction after rectal cancer treatment, explore sex‐based reporting disparities, and identify priorities for inclusive, patient‐centred research.

**Review Question(s):**

1. What are the differences in the reporting and assessment of sexual dysfunction between male and female participants after rectal cancer treatments?
2. What domains of sexual dysfunction are assessed by the Patient‐Reported Outcome Measures (PROMs) used in these studies?

## METHODS

This systematic review and narrative synthesis was carried out in accordance with the Preferred Reporting Items for Systematic Reviews and Meta‐Analyses (PRISMA) guidelines, following a protocol that was developed and approved by all authors in advance. The protocol was prepared a priori in line with the PRISMA‐P recommendations and registered with PROSPERO (International Prospective Register of Systematic Reviews) (CRD420251178502).

### Data selection

Eligibility was defined using the PICOS framework.

*Population*: Adult patients (≥18 years) diagnosed and who underwent treatment for rectal cancer. *Intervention*: Rectal cancer treatments, including but not limited to surgery, radiotherapy, chemotherapy.

*Comparisons*: Studies were deemed eligible independently of head‐to‐head comparisons. *Outcomes*: Studies were eligible if they reported sexual function outcomes using validated or clearly described PROMs. This included International Index of Erectile Function (IIEF) and/or the Female Sexual Function Index (FSFI), as well as broader cancer‐related QoL instruments where sexual function domains or items were extractable. IIEF and FSFI were included because they were the most commonly used sexual function‐specific PROMs in the rectal cancer literature. As included studies reported outcomes by male/female classification rather than gender identity, we use “sex‐based” terminology, with “gender” retained only for broader contextual factors.

*Study design*: Eligible study designs included randomised controlled trials, prospective or retrospective cohort studies, comparative observational studies, cross‐sectional studies, and non‐comparative observational cohorts where sexual function outcomes were systematically assessed using validated or clearly described PROMs. Studies were excluded if they met at least one of the following criteria: (a) included patients less than 18 years, (b) included colon or anal cancer without clear distinction of rectal cancer patients, (c) focused on palliative care or non‐curative treatments, (d) not published in English, (e) case reports, case series, letters, editorials, conference abstracts, commentaries, and reviews, (f) full‐text unavailable, (g) secondary research, such as systematic reviews or meta‐analyses. In cases of overlapping populations between studies, the study with the largest sample size and longest follow‐up was included.

The initial search covered records from the last 30 years (March 14, 1995–March 14, 2025). An updated search was conducted prior to submission until March 25, 2026. Studies were assessed for eligibility through searches of the PubMed/MEDLINE, EMBASE, Scopus, CINAHL Plus, PsycINFO, and Cochrane Library databases. The pre‐defined complete search strategy is available in File S1.

### Data extraction

Two reviewers (VL, JW) independently extracted data using a standardised form, piloted on a subset of studies. Disagreements were resolved with a third reviewer (JC). Extracted data included study characteristics, demographics, treatment, follow‐up and PROM outcomes. Corresponding authors were contacted for missing relevant data.

### Quality assessment

Methodological quality was independently assessed by VL and JW using RoB 2 for randomised trials and MINORS for observational studies (File S2). The first eight MINORS items were applied to non‐comparative studies and all 12 to comparative studies. Scores were classified as high (≥13/16 or ≥19/24), moderate (9–12/16 or 13–18/24), or low (≤8/16 or ≤12/24) quality. Adequate follow‐up was pre‐specified as 12 months postoperatively. Certainty was assessed narratively according to risk of bias, consistency, directness, precision and reporting completeness. Response and PROM‐completion rates, where reported, informed interpretation; low or unreported completion was considered a potential source of response bias.

### Data analysis

A narrative synthesis of sexual dysfunction outcomes was performed to present the findings of studies, following the European Social Research Council Guidance on the Conduct of Narrative Synthesis in Systematic Reviews and the Synthesis Without Meta‐analysis (SWiM) guideline [25, 26]. Data were summarised using numbers and percentages, where appropriate. Studies were grouped by PROM type, sex‐specific reporting, treatment modality, and timing of outcome assessment, with synthesis focused on patterns of reporting, direction of findings, and completeness of sex‐specific outcome data rather than pooled effect estimates.

## RESULTS

### Study selection

**FIGURE 1:**
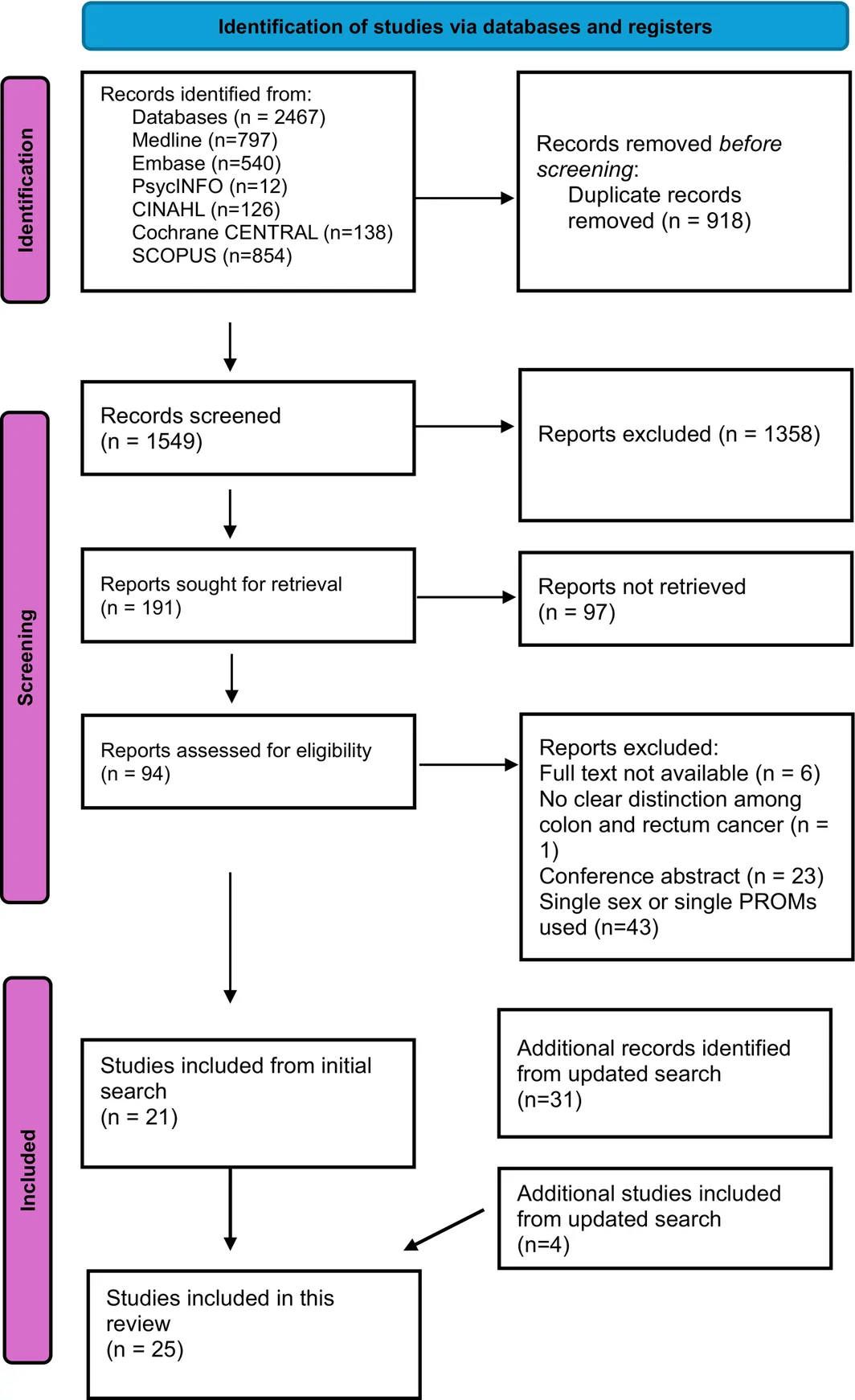
Flowchart depicting study selection.

A total of 2,467 records were identified. After removing 918 duplicate records, 1,549 records were screened based on titles and abstracts. Of these, 1,358 records were excluded. Ninety‐four full‐text articles were assessed for eligibility. Following full‐text review, 21 studies met the inclusion criteria (Figure 1). The updated search from March 14, 2025, to March 25, 2026, yielded 31 records, and four were eligible, giving a final total of 25 included studies (Figure 1).

### Study characteristics

A total of 25 studies comprising 4,071 patients were included, with sample sizes ranging from 40 to 948 participants. Mean age ranged from 53 to 66 years, and 65.8% were male. Twelve studies were prospective, 11 retrospective, and two randomised controlled trials. Six were multicentre studies. Follow‐up ranged from weeks to 4 years. Study characteristics and quality assessments are presented in Tables 1, 2, 3.

**TABLE 1:** Demographics of the included studies.

Author(s)	Year	Country	Study setting	Study design	Study period (yyyy or mm/yyyy)	No. of patients	Age		Follow‐up	Timepoints	Response rate	
							Mean	Median			Male	Female
Acquati et al.	2022	USA	MC	PS	08/2014–12/2017	40	53.2	NR	NR	B, PR, PC, 4w	85%	85%
Adam et al.	2016	UK	SC	PS	2008–2012	169	NR	61	12 m	B, PR, 3 m, 12 m	NR	NR
Attaallah et al.	2014	Turkey	SC	RS	01/2012–09/2013	84	NR	60	NR	Single survey	NR	NR
Au et al.	2012	Taiwan	SC	RS	07/2008–01/2009	120	60.8	NR	NR	Single survey	NR	NR
Bjoern et al.	2022	Denmark	MC	PS	TaTME: 08/2016–04/2019 LaTME/RoTME: 01/2011–09/2014	207	NR	TaTME: 69 LaTME/RoTME: 67.5	TaTME: 12 m LaTME/RoTME: 6 m	TaTME: B, 3 m, 12 m LaTME/RoTME: B, 30d, 6 m	28.3%	18.3%
Dulskas et al.	2016	Lithuania	SC	PS	01/2008–12/2014	54	60.04	NR	6 m	B, 6 m	NR	NR
Duran et al.	2015	Turkey	SC	PS	08/2008–12/2010	56	NR	NR	4 m	B, 3 m	NR	NR
Grass et al.	2021	Germany, Italy, Taiwan	MC	PS	01/2014–02/2018	120	62.9	NR	12 m	12 m	NR	NR
Gurbanov et al.	2025	Turkey	SC	RS	03/2017–12/2021	100	Ro: 58.09 Lap: 60.55	NR	Ro: 12.77 m Lap: 30.68 m	3 m, 6 m, 12 m	NR	NR
Hendren et al.	2005	Canada	SC	RS	1980–2003	180	NR	60	NR	Single survey	80.5%	81%
Kim et al.	2018	Korea	SC	RS	2009–2013	260	60.25	NR	NR	B, 3 m, 6 m, 12 m	NR	NR
Laohawiriyakamol et al.	2022	Thailand	SC	PS	01/2014–12/2016	91	NR	NR	12 m	B, 3 m, 12 m	NR	NR
Li et al.	2021	China	SC	PS	01/2012–08/2019	948	53.7	NR	12 m	B, 12 m	NR	NR
Liu et al.	2022	China	SC	PS	01/2017–01/2020	135	58.68	NR	12 m	B, 1 m, 3 m, 6 m, 12 m	NR	NR
Liu et al.	2025	China	SC	RS	01/2020–03/2023	235	Ro: 63 Lap: 64	NR	24 m	B, 3 m, 6 m, 12 m, 24 m	68.4%	68.8%
Macháčková et al.	2022	Czech Republic	SC	RS	03/2016–06/2018	66	61.5	NR	24 m	B, 3 m, 6 m, 12 m, 24 m	NR	NR
Mercieca‐Bebber et al.	2023	Australia	MC	RCT	2010–2014	473	NR	65	12 m	B, 4‐6w, 3 m, 12 m	85.2%	90.1%
Numata et al.	2025	Japan	MC	PS	01/2014–03/2017	135	63.6; 62.3	NR	12 m	B, 3 m, 6 m, 12 m	NR	NR
Pang et al.	2020	USA	SC	RS	2005–2016	47	NR	NR	NR	Single survey	NR	NR
Pontallier et al.	2016	France	SC	RCT	06/2008–02/2012	72	NR	62	12 m	B, 12 m	NR	NR
Rooney et al.	2024	USA	SC	RS	01/2017–12/2020	47	NR	NR	NR	Single survey	NR	NR
Sharabiany et al.	2022	Netherlands and UK	MC	RS	Netherlands: 2001–2018 UK: 2010–2018	125	64	NR	NR	B, Post‐op	NR	NR
Torrijo et al.	2021	Spain	SC	PS	01/2017–03/2019	80	66.36	NR	12 m	B, 6 m, 12 m	NR	NR
Tschann et al.	2022	Austria	SC	RS	01/1998–12/2020	79	NR	76	NR	Single survey	NR	NR
Wong et al.	2025	China	SC	PS	01/2016–12/2021	148		Ro: 67.5, Ta: 64.5	12 m	1 m, 3 m, 6 m, 12 m	96.5%	NR
					Total	4,071						

Abbreviations: #, Number; B, Baseline; LaTME, Laparoscopic Total Mesorectal Excision; m, month(s); MC, Multicentre; NR, Not reported; PC, Post Chemotherapy; PR, Post Radiotherapy; PS, Prospective Study; RCT, Randomised Controlled Trial; Ro, Robotic; RoTME, Robotic Total Mesorectal Excision; RS, Retrospective Study; SC, Single Centre; Ta, Transanal; TaTME, Transanal Total Mesorectal Excision; w, week(s).

**TABLE 2:** Quality assessment of the included non‐randomised studies using the Modified Methodological Items for Non‐Randomised Studies (MINORS).

Study	1	2	3	4	5	6	7	8		9	10	11	12	Total	Quality
Acquati et al. [27]	**2**	**1**	**2**	**2**	**1**	**1**	**0**	**0**	Additional criteria in ccc coo comparative study					9/16	Moderate
Adam et al. [28]	**2**	**2**	**2**	**2**	**1**	**2**	**0**	**0**						11/16	Moderate
Attaallah et al. [29]	**2**	**1**	**1**	**2**	**1**	**0**	**0**	**0**						7/16	Low
Au et al. [30]	**2**	**1**	**1**	**2**	**1**	**0**	**1**	**2**						10/16	Moderate
Bjoern et al. [31]	**1**	**2**	**2**	**2**	**1**	**2**	**0**	**0**		**2**	**1**	**1**	**1**	13/24	Moderate
Dulskas et al. [32]	**2**	**1**	**2**	**2**	**1**	**1**	**0**	**0**						9/16	Moderate
Duran et al. [33]	**2**	**2**	**2**	**2**	**1**	**1**	**2**	**2**						14/16	High
Grass et al. [34]	**2**	**2**	**2**	**2**	**1**	**2**	**1**	**0**		**2**	**2**	**1**	**2**	19/24	High
Gurbanov et al. [35]	**2**	**1**	**0**	**2**	**1**	**2**	**0**	**0**		**2**	**2**	**1**	**2**	15/24	Moderate
Hendren et al. [15]	**2**	**1**	**0**	**2**	**1**	**2**	**0**	**0**						8/16	Low
Kim et al. [36]	**2**	**2**	**2**	**2**	**1**	**2**	**1**	**0**		**2**	**2**	**2**	**2**	20/24	High
Laohawiriyakamol et al. [37]	**2**	**2**	**2**	**2**	**1**	**2**	**1**	**0**						12/16	Moderate
Li et al. [38]	**2**	**2**	**2**	**2**	**2**	**2**	**1**	**0**						13/16	High
Liu et al. [39]	**2**	**2**	**2**	**2**	**1**	**2**	**2**	**2**		**2**	**2**	**2**	**2**	23/24	High
Liu et al. [40]	**2**	**1**	**0**	**2**	**1**	**2**	**0**	**0**		**2**	**2**	**1**	**2**	15/24	Moderate
Macháčková et al. [41]	**2**	**2**	**2**	**2**	**1**	**2**	**1**	**0**		**2**	**2**	**2**	**1**	19/24	High
Numata et al. [42]	**2**	**2**	**2**	**2**	**1**	**2**	**1**	**0**		**2**	**2**	**1**	**1**	18/24	Moderate
Pang et al. [43]	**2**	**1**	**0**	**2**	**1**	**2**	**0**	**0**						8/16	Low
Rooney et al. [44]	**2**	**1**	**0**	**2**	**1**	**2**	**0**	**0**		**2**	**2**	**2**	**1**	15/24	Moderate
Sharabiany et al. [45]	**2**	**1**	**0**	**2**	**1**	**2**	**0**	**0**						8/16	Low
Torrijo et al. [46]	**2**	**2**	**2**	**2**	**1**	**2**	**1**	**0**						12/16	Moderate
Tschann et al. [47]	**2**	**1**	**0**	**2**	**1**	**2**	**0**	**0**						8/16	Low
Wong et al. [48]	**2**	**2**	**1**	**2**	**1**	**2**	**0**	**0**		**2**	**2**	**2**	**1**	17/24	Moderate

*Note*: MINORS items 1–8 apply to all studies, while items 9–12 apply only to comparative studies; therefore, the maximum possible score is 16 for non‐comparative studies and 24 for comparative studies. Each item is scored as 0 = not reported, 1 = reported but inadequate, and 2 = reported and adequate. Colour coding: green = 2, yellow = 1, and red/pink = 0. For the overall quality classification, green, yellow, and red/pink indicate high, moderate, and low methodological quality, respectively.

**TABLE 3:** Risk of bias (RoB 2) assessment for randomised controlled trials.

Study (year)	Domain 1	Domain 2	Domain 3	Domain 4	Domain 5	Overall risk
Mercieca‐Bebber et al. [49]	Low	Low	Low	Some	Low	Some
Pontallier et al. [50]	Low	Some	Low	Some	Low	Some

*Note*: RoB 2 judgements are abbreviated in the table as Low, Some, and High. These correspond to the official RoB 2 categories “Low risk of bias”, “Some concerns”, and “High risk of bias”, respectively. Domain 1: Randomisation process; Domain 2: Deviations from intended interventions; Domain 3: Missing outcome data; Domain 4: Measure of the outcome; Domain 5: Selection of the reported results Color coding: green = low risk of bias, yellow = some concerns, and red/pink = high risk of bias.

### Narrative synthesis

Due to the considerable heterogeneity among the included studies, a quantitative meta‐analysis was not feasible. Therefore, a narrative synthesis was undertaken to summarise the findings.

### Domains of sexual dysfunction and PROMs used

**FIGURE 2:**
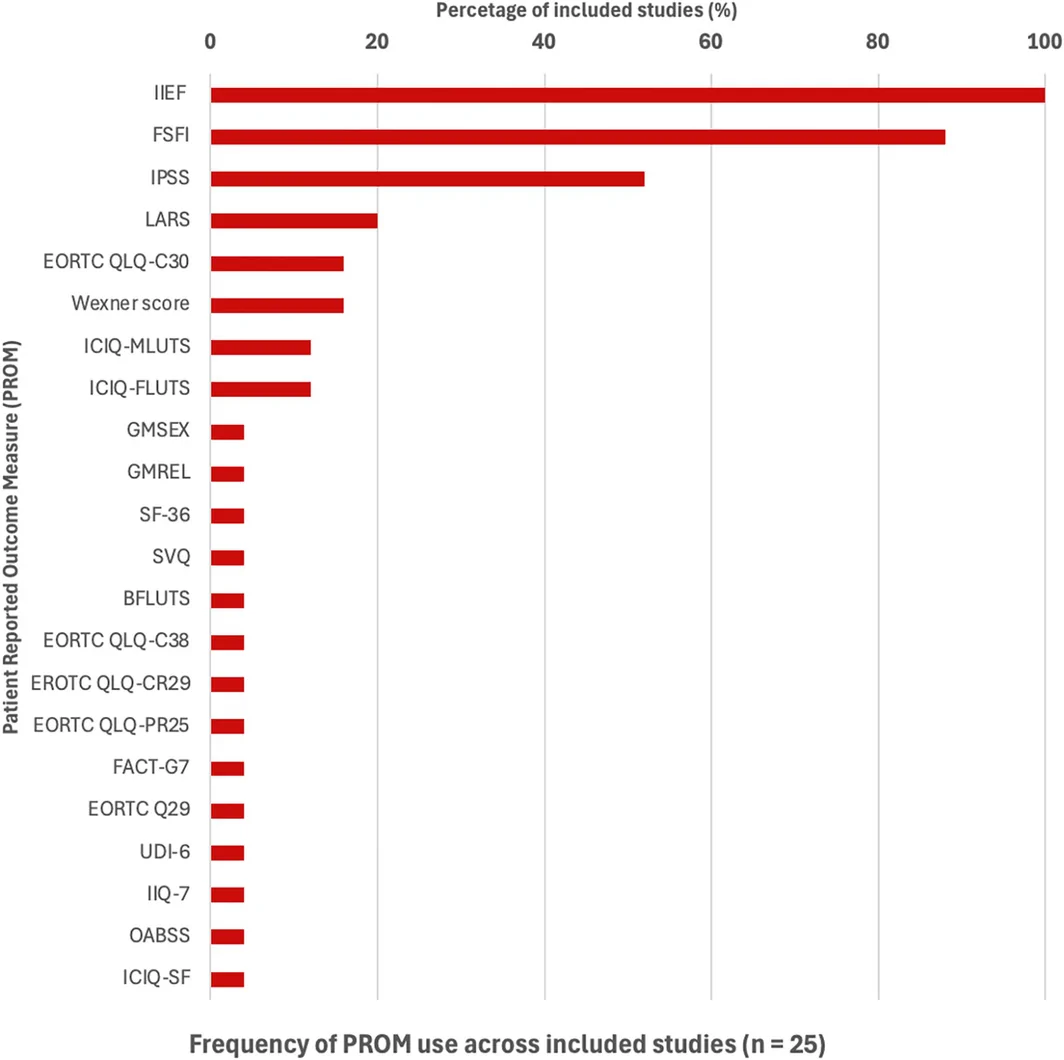
Frequency of patient‐reported outcome measure (PROM) use across included studies (*n* = 25). Bars represent the percentage of included studies using each PROM. Different versions of the IIEF and FSFI were grouped under their respective parent instruments.

Most studies incorporated validated PROMs, but instrument selection varied considerably (Figure 2), and these were utilised selectively or without domain‐specific reporting. Thirteen studies used the shorter versions of IIEF (IIEF‐5, a five‐item instrument) [29, 36, 37, 38, 39, 40, 41, 42, 44, 46, 47, 48, 50], which primarily assesses erectile function with one item relating to intercourse satisfaction, compared to IIEF‐15, which assesses multiple domains such as orgasmic function, sexual desire, or overall satisfaction. For female sexual health, different or adapted versions of FSFI were used. Six studies used FSFI‐6 [28, 31, 37, 38, 47, 50], with one study using FSFI‐9 [32], IFSF [33], IFSI [29], and the rest of the studies used FSFI‐19.

Additional PROMs were used to assess urinary or bowel function, including the International Prostate Symptom Score (IPSS) [31, 32, 33, 34, 35, 36, 37, 39, 40, 41, 42, 48, 50], Bristol Female Lower Urinary Tract Symptoms (BFLUTS) [32], Low Anterior Resection Syndrome (LARS) score [31, 34, 35, 47, 50], International Consultation on Incontinence Questionnaire—Male Lower Urinary Tract Symptoms (ICIQ‐MLUTS) [31, 34, 44], and International Consultation on Incontinence Questionnaire—Female Lower Urinary Tract Symptoms (ICIQ‐FLUTS) [31, 34, 44]. Others used broad quality‐of‐life measures such as EORTC QLQ‐C30 [31, 36, 47], EORTC QLQ‐CR29 [45, 49], EORTC QLQ‐CR38 [15], or SF‐36 [31], which contain limited sexual health items.

### Sex representation

**FIGURE 3:**
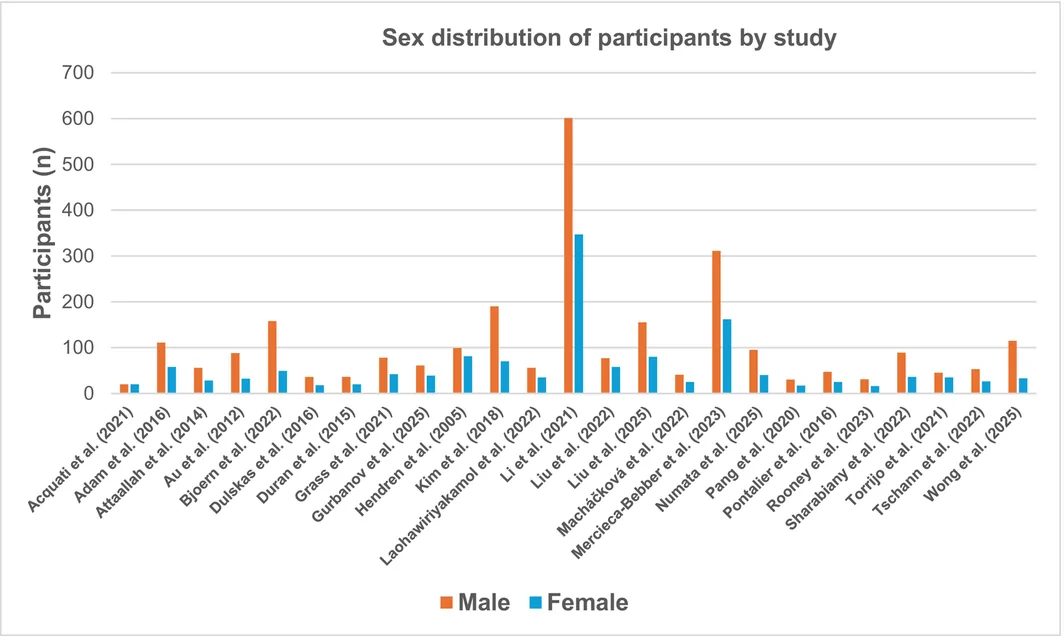
Sex distribution by study.

Sex representation imbalance was evident across almost all studies. Participants were predominantly male (2,679/4,071; 65.8%) with females representing 1,392/4,071 (34.2%) (Figure 3). At the study level, female representation ranged from 22% to 50% (median 35%), although the upper bound (50%) was observed in one study [27] where participants were enrolled as heterosexual couples, resulting in balanced sex distribution by study design; in the remaining studies, women comprised approximately one‐quarter to one‐third of participants. The proportion of male participants ranged from 50% to 78%, with six studies reporting >70% male population predominance [30, 31, 36, 42, 45, 48].

### Comparability and assessment

Most studies incorporated a baseline assessment (B) with follow‐up clustered around the first postoperative year, commonly at 3 months (3 m) and 12 months (12 m) [28, 36, 37, 39, 40, 41, 48, 49]; two studies included earlier post‐treatment assessments such as post‐radiotherapy (PR) and post‐chemotherapy (PC) [27, 28]. One study reported procedure‐specific follow‐up schedules, using different timepoints depending on the operative approach [31]. Six studies used single cross‐sectional surveys without repeated measurements [15, 29, 30, 43, 44, 47]. Stratified subgroup analyses were reported in four studies based on surgical intervention [34, 36, 41, 50], and [44] based on stoma status. Sexual activity status, sexual preference, and menopausal status were rarely reported.

### Reporting disparities

Patient‐reported outcome measures (PROMs) reporting was inconsistent, and overall domain granularity was uncommon across studies, with a clear skew towards reporting total scores rather than detailed domains. Fourteen studies reported total scores only [29, 33, 35, 36, 37, 38, 39, 40, 41, 42, 44, 47, 48, 50], without domain‐specific details. Eight studies reported both total and domain‐level results [15, 27, 30, 31, 32, 34, 43, 45], while only three provided domain‐level reporting [28, 46, 49]. All studies reported male and female sexual function separately, except [36, 42, 48], which restricted sexual function assessment and analysis to male patients only. Thirteen studies reported adjusted analyses accounting for covariates, including two propensity score‐matched analyses [36, 48]. Of these, two studies [28, 37] restricted adjusted modelling to the male cohort, with female sexual outcomes not included in multivariable analyses and no clear justification provided.

Six studies [15, 27, 31, 40, 48, 49] reported on response rates. Only one study reported sexual function‐related PROMs filling rate [40]. For studies with repeated time points, response rates at each follow‐up assessment were not reported in any studies. Methods for handling missing PROMs data (e.g., complete‐case analysis or imputation) were not reported.

Eight studies placed greater reporting emphasis on male outcomes [31, 36, 37, 42, 43, 46, 48, 50]. Small female sample size was reported in 16/25 studies [28, 29, 30, 32, 33, 34, 37, 38, 42, 43, 44, 45, 46, 47, 48, 50]. Among these, two studies explicitly noted that women were under‐represented [30, 34], and four studies reported reluctance among female participants to complete sexual function PROMs [28, 37, 38, 50].

## DISCUSSION

This systematic review of 25 studies involving 4,071 patients found that sexual function outcomes after multimodal rectal cancer treatment were assessed inconsistently and reported with clear sex‐based disparities. The evidence base was male‐predominant and often male‐oriented, with sexual outcomes most consistently reported for men, particularly erectile and ejaculatory function. In contrast, female sexual function was commonly condensed to overall scores, and in some studies, the female cohort was excluded from sexual outcome analyses or multivariable models without justification [28, 36, 37, 42, 48]. These findings suggest that the current survivorship evidence disproportionately reflects male sexual outcomes and may provide a less complete account of female sexual health after rectal cancer treatment. This contrasts with survivorship guidance (NCCN, ASCO, Macmillan Cancer Support) that advocates proactive, routine and inclusive assessment of sexual health [9, 17, 18, 19].

These findings are best interpreted as sex‐based reporting disparities rather than evidence of systematic bias. The review did not assess causal mechanisms or author intent, and the observed disparities may reflect smaller female samples, lower PROM completion, or broader methodological limitations.

### Under‐representation and selective inclusion

Women constituted approximately one‐third of participants overall, and many studies (16/25) explicitly reported small female samples, limiting statistical power for sex‐based comparisons. However, limited sample size alone does not fully explain the reporting gap. Even when women were included, female sexual outcomes were frequently presented with less detail than male outcomes, and adjusted modelling was sometimes restricted to men. Five studies omitted sexual function analysis in women while including women in other QoL measures without clear justification. Contemporary incidence data show male predominance in rectal cancer globally and in UK bowel cancer statistics [1]; therefore, the lower female proportion in this review may partly reflect epidemiology, although limited female‐specific PROM use, reduced domain‐level reporting, and exclusion from some analyses suggest that epidemiology alone does not explain the reporting gap. This pattern aligns with broader survivorship literature in colorectal and pelvic cancers, where female sexual dysfunction is often described as comparatively under‐studied, variably defined, and supported by less developed evidence for targeted interventions [51, 52, 53, 54], leaving female outcomes less certain and less comparable. There is no data reported on sexual orientation as part of these studies, which may have relevance when considering use of the FSFI score, which specifically defines sexual intercourse as “penile penetration (entry) of the vagina” and could impact on response rates.

### Limited female domain granularity and measurement limitations

A further consistent finding was reliance on the IIEF, particularly IIEF‐5, which largely emphasises erectile and ejaculatory function. Female outcomes were assessed using a range of FSFI versions and adaptations such as FSFI‐6, FSFI‐9, FSFI‐19, IFSF, and IFSI, but were commonly reported as total scores rather than domain‐level results. The lack of domain granularity in female reporting limits interpretability, as clinically relevant domains (e.g., desire, arousal, lubrication, orgasm, satisfaction, and dyspareunia) may be differentially affected by pelvic surgery, radiotherapy, stoma status, and treatment‐related body image changes [55, 56, 57]. Reed et al. [58] highlighted the appropriateness of the FSFI tool and emphasised the need for consistent use of validated instruments, while acknowledging that thresholds used to define sexual dysfunction may need to vary by menopausal status (e.g., younger women versus peri‐ or post‐menopausal women). More detailed domain‐level reporting could help identify symptom patterns and support targeted counselling and interventions [59].

International Index of Erectile Function (IIEF) and/or Female Sexual Function Index (FSFI) were the most frequently used sexual function‐specific PROMs, but both are generic instruments and were not developed specifically for cancer populations. They may therefore miss rectal cancer‐specific concerns, including stoma‐related body image, bowel dysfunction during intimacy, radiotherapy effects, relationship context, and altered sexual practices. Cancer‐related QoL tools were also included where sexual function items were extractable, but these were reported inconsistently and often provided limited sexual health detail.

### Missing contextual factors

Key contextual determinants of sexual function were rarely reported, including baseline sexual activity, sexual practices and preferences, sexual orientation, and menopausal status. This limits interpretation for both sexes and is particularly important for women, in whom menopausal transition and treatment‐induced ovarian dysfunction may substantially influence symptoms [17, 60]. The included studies did not directly assess whether relationship changes, reduced or avoided intercourse, or stoma‐related concerns influenced PROM scores; these therefore remain possible explanations. Poor reporting of sexual practices and preferences also limits inclusivity, as many PROMs assume heterosexual, intercourse‐based activity and may not reflect sexual wellbeing across diverse orientations and behaviours [58, 59, 60]. Reasons for PROM non‐completion were also rarely investigated. Embarrassment, reduced sexual activity, relationship changes, and symptom severity are plausible explanations, but their contribution cannot be determined from the available evidence. Heterogeneous follow‐up schedules and incomplete reporting of response rates and missing‐data methods further reduce comparability. Transparent reporting of contextual variables, follow‐up completeness, PROM completion, and missing‐data methods is, therefore, essential.

### Clinical implications

Current evidence may shape survivorship counselling in ways that prioritise male erectile outcomes while under‐addressing female symptoms such as dyspareunia, vaginal dryness, arousal difficulties, and reduced satisfaction. Routine clinical pathways should incorporate proactive, inclusive sexual health discussions, with appropriate referral pathways (e.g., psychosexual services, pelvic floor physiotherapy, menopause services, and counselling). Importantly, limited evidence for women should not be interpreted as evidence of lower symptom burden, particularly when measurement and participation data are incomplete.

### Strength and limitations

This review included diverse study designs and settings and focused on sex‐based reporting disparities. However, heterogeneity in PROMs, outcome definitions, follow‐up, and study quality limited synthesis. Available primary data rarely captured sexual activity, menopausal status, or relationship context. Overall certainty was low because of methodological heterogeneity, incomplete response‐rate reporting, and limited sex‐specific outcome data.

### Future directions

Future rectal cancer survivorship studies should:

1. Prioritise equitable recruitment and retention of women;
2. Investigate strategies to improve the participation of women in studies assessing sexual function;
3. Record and report contextual factors, including sexual activity, sexual practices and preferences, menopausal status, and sexual orientation.
4. Develop a core outcome set for female sexual function after rectal cancer treatment to reduce PROM heterogeneity and improve the consistency, comparability, and clinical relevance of future reporting.

These steps are necessary to develop an evidence base that accurately reflects the sexual health needs of all patients after rectal cancer treatment.

## CONCLUSION

Sexual function outcomes after rectal cancer treatment are inconsistently reported, with low certainty due to heterogeneous study design, PROMs selection, follow‐up timing, response rates, and incomplete sex‐specific reporting. Although the lower proportion of female participants may partly reflect the male predominance of rectal cancer, limited female‐specific PROMs use and domain‐level reporting suggest that epidemiology alone does not explain the existing reporting gap. Future research should use standardised, sex‐specific and cancer‐relevant outcomes, with development of a core outcome set for female sexual function after rectal cancer treatment.

## AUTHOR CONTRIBUTIONS

**Jared Torkington:** Writing – review and editing; supervision. **Julie A. Cornish:** Conceptualization; writing – review and editing; methodology; supervision; resources; investigation; data curation. **Vignesh Lakshmanan:** Conceptualization; investigation; writing – original draft; methodology; validation; writing – review and editing; formal analysis; data curation; project administration; resources. **Jennifer Waterman:** Investigation; writing – review and editing; data curation. **Alun Meggy:** Writing – review and editing.

## FUNDING INFORMATION

No specific funding was received for this work.

## CONFLICT OF INTEREST STATEMENT

The authors declare no conflicts of interest.

## ETHICS STATEMENT

This study was a systematic review and narrative synthesis of previously published literature.

## CLINICAL TRIAL REGISTRATION

This review was registered with PROSPERO (CRD420251178502).

## Supporting information


**File S1.** Search syntax.

**File S2.** Modified Methodological Items for Non‐Randomised Studies (MINORS) tool—Adapted for this systematic review.

## Data Availability

The data that support the findings of this study are available from the corresponding author upon reasonable request.

## References

[1] Bowel Cancer Incidence Statistics | Cancer Research UK [Internet]. [cited 2026 Mar 26]. Available from: https://www.cancerresearchuk.org/health‐professional/cancer‐statistics/statistics‐by‐cancer‐type/bowel‐cancer/incidence

[2] Halfter K , Schubert‐Fritschle G , Röder F , Kim M , Werner J , Belka C , et al. Advances in rectal cancer: real‐world evidence suggests limited gains in prognosis for elderly patients. Cancer Epidemiol. 2023;86:102440. 10.1016/j.canep.2023.102440 37572415

[3] Zeineddine FA , Zeineddine MA , Yousef A , Gu Y , Chowdhury S , Dasari A , et al. Survival improvement for patients with metastatic colorectal cancer over twenty years. NPJ Precis Oncol. 2023;7(1):16. 10.1038/s41698-023-00353-4 36781990 PMC9925745

[4] Buchwald P , Hall C , Davidson C , Dixon L , Dobbs B , Robinson B , et al. Improved survival for rectal cancer compared to colon cancer: the four cohort study. ANZ J Surg. 2018;88(3):E114–E117. 10.1111/ans.13730 27618786

[5] Rocha HB , Carneiro BC , Vasconcelos PA , Pereira R , Quinta‐Gomes AL , Nobre PJ . Promoting sexual health in colorectal cancer patients and survivors: results from a systematic review. Healthcare (Basel). 2024;12(2):253. 10.3390/healthcare12020253 38275533 PMC10815307

[6] Feier CVI , Paunescu IA , Faur AM , Cozma GV , Blidari AR , Muntean C . Sexual functioning and impact on quality of life in patients with early‐onset colorectal cancer: a systematic review. Diseases. 2024;12(4):66. 10.3390/diseases12040066 38667524 PMC11049269

[7] Jung WB . Beyond survival: a comprehensive review of quality of life in rectal cancer patients. Ann Coloproctol. 2024;40(6):527–537. 10.3393/ac.2024.00745.0106 39748551 PMC11701447

[8] Panjari M , Bell RJ , Burney S , Bell S , McMurrick PJ , Davis SR . Sexual function, incontinence, and wellbeing in women after rectal cancer—a review of the evidence. J Sex Med. 2012;9(11):2749–2758. 10.1111/j.1743-6109.2012.02894.x 22905761

[9] Denlinger CS , Barsevick AM . The challenges of colorectal cancer survivorship. J Natl Compr Canc Netw. 2009;7(8):883–893; quiz 894. 10.6004/jnccn.2009.0058 19755048 PMC3110673

[10] Roussin M , Lowe J , Hamilton A , Martin L . Sexual quality of life in young gynaecological cancer survivors: a qualitative study. Qual Life Res. 2023;32(7):2107–2115. 10.1007/s11136-023-03386-1 36947327 PMC10241684

[11] Hansen SB , Oggesen BT , Fonnes S , Rosenberg J . Erectile dysfunction is common after rectal cancer surgery: a cohort study. Curr Oncol. 2023;30(10):9317–9326. 10.3390/curroncol30100673 37887573 PMC10605730

[12] Schover LR , van der Kaaij M , van Dorst E , Creutzberg C , Huyghe E , Kiserud CE . Sexual dysfunction and infertility as late effects of cancer treatment. EJC Suppl. 2014;12(1):41–53. 10.1016/j.ejcsup.2014.03.004 26217165 PMC4250536

[13] Kim JY , Kim NK , Lee KY , Hur H , Min BS , Kim JH . A comparative study of voiding and sexual function after Total Mesorectal excision with autonomic nerve preservation for rectal cancer: laparoscopic versus robotic surgery. Ann Surg Oncol. 2012;19(8):2485–2493. 10.1245/s10434-012-2262-1 22434245

[14] Lee C , Park IJ . Sex disparities in rectal cancer surgery: an in‐depth analysis of surgical approaches and outcomes. World J Mens Health. 2024;42(2):304–320. 10.5534/wjmh.230335 38449456 PMC10949018

[15] Hendren SK , O'Connor BI , Liu M , Asano T , Cohen Z , Swallow CJ , et al. Prevalence of male and female sexual dysfunction is high following surgery for rectal cancer. Ann Surg. 2005;242(2):212–223. 10.1097/01.sla.0000171299.43954.ce 16041212 PMC1357727

[16] Averyt JC , Nishimoto PW . Addressing sexual dysfunction in colorectal cancer survivorship care – Averyt. J Gastrointest Oncol. 2014;5(5):388–394. [Internet]. [cited 2026 Mar 26]. Available from: https://jgo.amegroups.org/article/view/3006/html 25276411 10.3978/j.issn.2078-6891.2014.059 PMC4173048

[17] Cancer and your sex life. [Internet]. [cited 2026 Apr 4]. Available from: https://www.macmillan.org.uk/cancer‐information‐and‐support/treatment/coping‐with‐treatment/your‐sex‐life

[18] Denlinger CS , Sanft T , Baker KS , Baxi S , Broderick G , Demark‐Wahnefried W , et al. Survivorship, version 2.2017. J Natl Compr Canc Netw. 2017;15(9):1140–1163. 10.6004/jnccn.2017.0146 28874599 PMC5865602

[19] Carter J , Lacchetti C , Andersen BL , Barton DL , Bolte S , Damast S , et al. Interventions to address sexual problems in people with cancer: American Society of Clinical Oncology clinical practice guideline adaptation of Cancer Care Ontario guideline. J Clin Oncol Off J Am Soc Clin Oncol. 2018;36(5):492–511. 10.1200/JCO.2017.75.8995 29227723

[20] Brissette V , Monton O , Demian M , Al Busaidi N , Moon J , Sabboobeh S , et al. Exploring patients' needs and expectations for information on sexual dysfunction after rectal cancer treatment: a qualitative study. Color Dis. 2024;26(8):1535–1543. 10.1111/codi.17048 38890007

[21] Pratt‐Chapman ML , Alpert AB , Castillo DA . Health outcomes of sexual and gender minorities after cancer: a systematic review. Syst Rev. 2021;10(1):183. 10.1186/s13643-021-01707-4 34154645 PMC8218456

[22] Boehmer U , Clark MA , Winter M , Berklein F , Ozonoff A . Sexual minority‐specific experiences of colorectal cancer survivors. Health Psychol. 2022;41(11):884–892. 10.1037/hea0001229 36074595 PMC10367941

[23] Doucette C , Milano MT , Kamen C . Patient perceptions of sexual orientation and gender identity data collection in an outpatient radiation oncology setting. Int J Radiat Oncol Biol Phys. 2023;116(1):68–78. A Red Journal Special Issue Health Equity, Diversity, and Inclusion, Part 1. 10.1016/j.ijrobp.2022.12.019 36549346

[24] Rossi A , Leone AG , Lambertini M , Sperti E , Cassani C , Vetromile A , et al. Sexual health in cancer care: a narrative review and position statement from the Italian Association of Medical Oncology (AIOM). ESMO Open. 2025;10(6):105311. 10.1016/j.esmoop.2025.105311 40505419 PMC12192329

[25] Popay J , Roberts H , Sowden A , Petticrew M , Arai L , Rodgers M , et al. Guidance on the Conduct of Narrative Synthesis in Systematic Reviews A Product from the ESRC Methods Programme. 2006 [cited 2026 Mar 26]. Available from: https://www.semanticscholar.org/paper/Guidance‐on‐the‐Conduct‐of‐Narrative‐Synthesis‐in‐A‐Popay‐Roberts/ed8b23836338f6fdea0cc55e161b0fc5805f9e27

[26] Campbell M , McKenzie JE , Sowden A , Katikireddi SV , Brennan SE , Ellis S , et al. Synthesis without meta‐analysis (SWiM) in systematic reviews: reporting guideline [Internet]. 2020. 10.1136/bmj.l6890 PMC7190266 31948937

[27] Acquati C , Hendren S , Wittmann D , Reese JB , Karam E , Duby A , et al. Psychological and sexual distress in rectal cancer patients and partners. Psychooncology. 2022;31(6):920–928. 10.1002/pon.5880 35001478

[28] Adam JP , Denost Q , Capdepont M , van Geluwe B , Rullier E . Prospective and longitudinal study of urogenital dysfunction after proctectomy for rectal cancer. Dis Colon Rectum. 2016;59(9):822–830. 10.1097/DCR.0000000000000652 27505110

[29] Attaallah W , Ertekin C , Tinay I , Yegen C . High rate of sexual dysfunction following surgery for rectal cancer. Ann Coloproctol. 2014;30(5):210–215. 10.3393/ac.2014.30.5.210 25360427 PMC4213936

[30] Au TY , Zauszniewski JA , King TM . Demographics, cancer‐related factors, and sexual function in rectal cancer patients in Taiwan: preliminary findings. Cancer Nurs. 2012;35(5):E17–E25. 10.1097/NCC.0b013e318233a966 22067695

[31] Bjoern MX , Clausen FB , Seiersen M , Bulut O , Bech‐Knudsen F , Jansen JE , et al. Quality of life and functional outcomes after transanal total mesorectal excision for rectal cancer—results from the implementation period in Denmark. Int J Color Dis. 2022;37(9):1997–2011. 10.1007/s00384-022-04219-2 35960389

[32] Dulskas A , Samalavicius NE . A prospective study of sexual and urinary function before and after total mesorectal excision. Int J Color Dis. 2016;31(6):1125–1130. 10.1007/s00384-016-2549-y 26960814

[33] Duran E , Tanriseven M , Ersoz N , Oztas M , Ozerhan IH , Kilbas Z , et al. Urinary and sexual dysfunction rates and risk factors following rectal cancer surgery. Int J Color Dis. 2015;30(11):1547–1555. 10.1007/s00384-015-2346-z 26264048

[34] Grass JK , Persiani R , Tirelli F , Chen CC , Caricato M , Pecorino A , et al. Robotic versus transanal total mesorectal excision in sexual, anorectal, and urinary function: a multicenter, prospective, observational study. Int J Color Dis. 2021;36(12):2749–2761. 10.1007/s00384-021-04030-5 PMC8589758 34537862

[35] Gurbanov V , Umman V , Bozbiyik O , Yoldas T . Comparative evaluation of near‐term oncologic, urinary, sexual, and postoperative outcomes in rectal cancer: laparoscopic vs. robotic approaches. Medicina (Mex). 2025;61(10):1726. 10.3390/medicina61101726 PMC12566341 41155713

[36] Kim HJ , Choi GS , Park JS , Park SY , Yang CS , Lee HJ . The impact of robotic surgery on quality of life, urinary and sexual function following total mesorectal excision for rectal cancer: a propensity score‐matched analysis with laparoscopic surgery. Color Dis. 2018;20(5):O103–O113. 10.1111/codi.14051 29460997

[37] Laohawiriyakamol S , Chewatanakornkul S , Wanichsuwan W , Ruangsin S , Sunpaweravong S , Bejrananda T . Urogenital dysfunction after laparoscopic surgery for rectal or sigmoid colon cancer. Asian J Surg. 2023;46(1):492–500. 10.1016/j.asjsur.2022.06.004 35717291

[38] Li K , He X , Tong S , Zheng Y . Risk factors for sexual dysfunction after rectal cancer surgery in 948 consecutive patients: a prospective cohort study. Eur J Surg Oncol. 2021;47(8):2087–2092. 10.1016/j.ejso.2021.03.251 33832775

[39] Liu Y , Liu M , Lei Y , Zhang H , Xie J , Zhu S , et al. Evaluation of effect of robotic versus laparoscopic surgical technology on genitourinary function after total mesorectal excision for rectal cancer. Int J Surg. 2022;104:106800. 10.1016/j.ijsu.2022.106800 35934282

[40] Liu D , Zhang Q , Guo F , Sun Z , Ren S . Robotic vs. 3D laparoscopic resection for rectal cancer: a single‐center retrospective study of short‐term outcomes and functional recovery. Front Surg. 2025;12:1630237. 10.3389/fsurg.2025.1630237 40822268 PMC12350314

[41] Macháčková M , Škrovina M , Szikhart M , Martínek L , Benčurik V , Bartoš J , et al. Urogenital dysfunction in patients after miniinvasive restorative low anterior resection with total mesorectal excision. Videosurg Miniinvasive Tech. 2022;17(3):506–514. 10.5114/wiitm.2022.116394 PMC9511913 36187065

[42] Numata M , Watanabe J , Tsukada Y , Suwa Y , Fukunaga Y , Hirano Y , et al. Patient‐reported outcomes and surgical results of hand‐sewn versus stapled anastomosis for lower rectal cancer located 4–5 cm from the anal verge: a subanalysis of the ultimate study. Ann Gastroenterol Surg. 2025;9(6):1215–1224. 10.1002/ags3.70063 41199980 PMC12586937

[43] Pang JH , Jones Z , Myers OB , Popek S . Long term sexual function following rectal cancer treatment. Am J Surg. 2020;220(5):1258–1263. 10.1016/j.amjsurg.2020.06.064 32680624

[44] Rooney MK , Pasli M , Chang GJ , Das P , Koay EJ , Koong AC , et al. Patient‐reported sexual function, bladder function and quality of life for patients with low rectal cancers with or without a permanent ostomy. Cancers (Basel). 2024;16(1):153. 10.3390/cancers16010153 PMC10778006 38201580

[45] Sharabiany S , Kreisel SI , Strijk GJ , Blok RD , Bosschieter J , Laan ETM , et al. Exploring the impact of urogenital organ displacement after abdominoperineal resection on urinary and sexual function. Int J Color Dis. 2022;37(10):2125–2136. 10.1007/s00384-022-04234-3 PMC9562368 36044045

[46] Torrijo I , Balciscueta Z , Tabet J , Martín MC , López M , Uribe N . Prospective study of sexual function and analysis of risk factors after rectal cancer surgery. Color Dis. 2021;23(6):1379–1392. 10.1111/codi.15589 33599035

[47] Tschann P , Weigl M , Brock T , Frick J , Sturm O , Presl J , et al. Identification of risk factors for sexual dysfunction after multimodal therapy of locally advanced rectal cancer and their impact on quality of life: a single‐center trial. Cancer. 2022;14(23):5796. 10.3390/cancers14235796 PMC9736520 36497279

[48] Wong PC , Chow FCL , Law WL , Foo CC . Anorectal and urogenital functional outcome after robotic and transanal total mesorectal excision for rectal cancer: a propensity score‐matched analysis. Tech Coloproctol. 2025;29(1):141. 10.1007/s10151-025-03172-w 40658258 PMC12259797

[49] Mercieca‐Bebber R , Eggins R , Brown K , Gebski VJ , Brewer K , Lai L , et al. Patient‐reported bowel, urinary, and sexual outcomes after laparoscopic‐assisted resection or open resection for rectal cancer: the Australasian laparoscopic cancer of the rectum randomized clinical trial (ALaCart). Ann Surg. 2023;277(3):449–455. 10.1097/SLA.0000000000005412 35166265

[50] Pontallier A , Denost Q , Van Geluwe B , Adam JP , Celerier B , Rullier E . Potential sexual function improvement by using transanal mesorectal approach for laparoscopic low rectal cancer excision. Surg Endosc. 2016;30(11):4924–4933. 10.1007/s00464-016-4833-x 26944728

[51] McCool‐Myers M , Theurich M , Zuelke A , Knuettel H , Apfelbacher C . Predictors of female sexual dysfunction: a systematic review and qualitative analysis through gender inequality paradigms. BMC Womens Health. 2018;18:108. 10.1186/s12905-018-0602-4 29929499 PMC6013982

[52] Prabhu SS , Hegde S , Sareen S . Female sexual dysfunction: a potential minefield. Indian J Sex Transm Dis AIDS. 2022;43(2):128–134. 10.4103/ijstd.IJSTD_82_20 36743096 PMC9890990

[53] Tsai TF , Yeh CH , Hwang TIS . Female sexual dysfunction: physiology, epidemiology, classification, evaluation and treatment. Urol Sci. 2011;22(1):7–13. 10.1016/S1879-5226(11)60002-X

[54] Weinberger JM , Houman J , Caron AT , Anger J . Female sexual dysfunction: a systematic review of outcomes across various treatment modalities. Sex Med Rev. 2019;7(2):223–250. 10.1016/j.sxmr.2017.12.004 29402732

[55] Drittone D , Specchia M , Mazzotti E , Mazzuca F . Sexual dysfunction in female rectal and anal cancer survivors: pathophysiology, clinical management, and integration into survivorship care. Cancer. 2025;17(19):3150. 10.3390/cancers17193150 PMC12523370 41097678

[56] Jensen PT , Froeding LP . Pelvic radiotherapy and sexual function in women. Transl Androl Urol. 2015;4(2):186–205. 10.3978/j.issn.2223-4683.2015.04.06 26816824 PMC4708128

[57] Forbes MK , Baillie AJ , Schniering CA . Critical flaws in the female sexual function index and the international index of erectile function. J Sex Res. 2014;51(5):485–491. 10.1080/00224499.2013.876607 24826876

[58] Reed SD , Carpenter JS , Larson J , Mitchell CM , Shifren J , Heiman J , et al. Toward a better measure of midlife sexual function: pooled analyses in nearly 1000 women participating in MsFLASH randomized trials. Menopause. 2022;29(4):397–407. 10.1097/GME.0000000000001940 35102098 PMC8976731

[59] Austria MD , Lynch K , Le T , Walters CB , Atkinson TM , Vickers AJ , et al. Sexual and gender minority persons' perception of the female sexual function index. J Sex Med. 2021;18(12):2020–2027. 10.1016/j.jsxm.2021.09.012 34732309 PMC8642307

[60] Lynch KA , Austria MD , Le T , Walters CB , Vickers A , Roche KL , et al. Suggestions for modifications to the female sexual function index based on cognitive interviews with sexual and gender minority individuals and cisgender, heterosexual persons. J Sex Med. 2023;20(6):871–877. 10.1093/jsxmed/qdad042 37057601 PMC10230643

