# Supplementary material for: Sexual dysfunction outcomes and reporting disparities after multimodal treatment for rectal cancer: A systematic review and narrative synthesis
Source: Colorectal Dis. 2026 Aug 28;28(9):e70599. doi: 10.1111/codi.70599 (PMC13525230; doi:10.1111/codi.70599)
Supplement: Supplementary file 1 [file CODI-28-0-s001.docx]

**Supplementary File 2. Modified Methodological Items for Non-Randomised Studies (MINORS) Tool – Adapted for this Systematic Review**

Each item is rated 0 (not reported), 1 (reported but inadequate), or 2 (reported and adequate). The global ideal score being 16 for non-comparative studies and 24 for comparative studies

| **Item** | **Sub-category question** | **2 = Adequate** | **1 = Inadequate** | **0 = Not reported** |
| --- | --- | --- | --- | --- |
| 1. Clearly stated aim | Was the study aim clearly stated and relevant to sexual or genitourinary function after rectal cancer treatment? | Explicit objective directly addresses sexual or genitourinary function after rectal cancer treatment, with gender-specific analysis or intent stated. | General QoL or functional outcomes aim with only indirect mention of sexual function. | No clear study aim or objectives or unrelated to sexual function. |
| 2. Inclusion of consecutive patients | Were all eligible rectal cancer patients consecutively included during the study period? | All eligible rectal cancer patients within a defined period included, with inclusion and exclusion criteria transparent. | Some criteria given but sampling appears selective, or incomplete. | Convenience or volunteer sample or unclear recruitment process. |
| 3. Prospective collection of data | Were sexual-function data collected prospectively according to a predefined protocol? | Sexual function data gathered prospectively based on pre-defined protocol. | Mix of prospective and retrospective data or unclear timeline. | Entirely retrospective medical records review. |
| 4. Endpoints appropriate to the aim | Were endpoints appropriate to the aim and measured using validated PROMs? | Primary endpoint clearly measures sexual function using validated PROMs (IIEF, FSFI). May have used additional PROMs (e.g., EORTC-QLQ-CR38 sexual items) | Sexual function mentioned but not primary endpoint or assessed using non-validated questions. | Endpoints unrelated to sexual function or unspecified. |
| 5. Unbiased assessment of endpoints | Were validated PROMs used consistently and applied equally to both sexes? | PROMs validated, self-administered, and applied equally to both sexes with consistent instructions. | Validated PROMs used but limited to one sex or inconsistent use across respondents. | Non-validated or clinician-interpreted, or obvious sex-specific bias. |
| 6. Follow-up period appropriate | Was the follow-up period sufficient (≥12 months) to capture late sexual dysfunction? | ≥12 months post-treatment or multiple timepoints capturing late sexual dysfunction. | <12 months or single short-term assessment. | No follow-up period stated or assessment cross-sectional only. |
| 7. Loss to follow-up <5% | Was loss to follow-up minimal and reported by sex? | ≥95% of eligible participants retained; attrition minimal and sex-specific losses described. | 5–20% loss or unclear sex distribution of non-responders. | >20% loss or follow-up or response rates not reported. |
| 8. Prospective calculation of study size | Was a sample-size or power calculation performed prospectively? | Power or sample-size calculation performed to detect differences in sexual-function outcomes. | Mention of expected enrolment without formal calculation. | No calculation; convenience sample. |
| **The next four items are additional criteria in the case of comparative studies.** | | | | |
| 9. Adequate control group | Was there an appropriate control group for comparison (e.g., TaTME vs RoTME)? | Comparison between clearly defined arms with clinical justification. | Comparative groups present but poorly matched or rationale unclear. | No true control or inappropriate comparison. |
| 10. Contemporary groups | Were groups treated during the same time period under similar conditions? | Both groups treated during the same period under similar institutional settings. | Overlapping but not identical time frames. | Historical control or data from different eras. |
| 11. Baseline equivalence of groups | Were the comparative groups equivalent at baseline? | Groups comparable in demographics, tumour stage, neoadjuvant therapy, and sex distribution. | Baseline variables reported but with imbalance not accounted for. | No baseline characteristics provided. |
| 12. Adequate statistical analyses | Were statistical analyses appropriate and adjusted for confounders? | Correct statistical tests used with adjustments for confounders (age, sex, treatment type, radiotherapy). | Basic statistics only, no confounder adjustment. | No or inappropriate analysis. |

QoL – Quality of Life; PROMs – Patient Reported Outcome Measures; IIEF – International Index of Erectile Function; FSFI – Female Sexual Function Index; EORTC QLQ-C38 - European Organisation for Research and Treatment of Cancer Quality-of-life Questionnaire Core 38; TaTME – Transanal Total Mesorectal Excision; RoTME – Robotic Total Mesorectal Excision.

**Interpretation:**

| **Total score range** | **Quality rating** | **Interpretation** |
| --- | --- | --- |
| ≥ 13 (non-comparative) / ≥ 19 (comparative) | High | Strong design, prospective, validated PROMs, balanced sex reporting. |
| 9 – 12 (non-comparative) / 13 – 18 (comparative) | Moderate | Acceptable design but with methodological or reporting gaps. |
| ≤ 8 (non-comparative) / ≤ 12 (comparative) | Low | Major weaknesses (retrospective data, poor follow-up, or unclear endpoints). |
