# Supplementary material for: Sexual dysfunction outcomes and reporting disparities after multimodal treatment for rectal cancer: A systematic review and narrative synthesis
Source: Colorectal Dis. 2026 Aug 28;28(9):e70599. doi: 10.1111/codi.70599 (PMC13525230; doi:10.1111/codi.70599)
Supplement: Supplementary file 2 [file CODI-28-0-s002.docx]

**Supplementary File 1:** Search Syntax

**Date searches carried out:** 14/03/2025

**Databases searched:**

Medline: 1946 – current (ALL) - Ovid

Embase: 1974 – current - Ovid

APA PsycINFO: 1806 – Current - Ovid

CENTRAL – Cochrane CENTRAL Register of Control Trials – Cochrane Collaboration

CINAHL Plus with Full text – 1937 – Current - Ebsco

Scopus – 1974 – current

**Medline search strategy:** Number of results before deduplication: 797

Ovid MEDLINE(R) ALL <1946 to March 12, 2025>

1	exp Rectal Neoplasms/	57134

2	((rectal or rectum* or anorectal) adj2 (cancer* or neoplasm* or adenocarcinoma* or lymphoma* or tumo?r* or sarcoma* or melanoma* or leiomyosarcoma*)).ti,ab.	39545

3	((colorectal adj2 (cancer* or neoplasm* or adenocarcinoma* or lymphoma* or tumo?r* or sarcoma* or melanoma* or leiomyosarcoma*)) and (rectal or rectum*)).ti,ab.	12880

4	1 or 2 or 3	74392

5	exp Sexual Dysfunction, Physiological/	35079

6	exp Sexual Dysfunctions, Psychological/	31729

7	exp Infertility/ or exp Fertility/	119448

8	orgasm/	2908

9	(Fecal incontinence/ or exp Urinary incontinence/) and (coitus/ or masturbation/ or orgasm/)	206

10	exp "Quality of Life"/ and (exp Infertility/ or exp Fertility/ or coitus/ or masturbation/ or orgasm/)	1418

11	((sexual or erectile or ejaculat* or orgasm* or arousal) adj (dysfunction* or function* or disorder* or health or impoten*)).ti,ab.	61847

12	((sexual adj (arousal or desire)) or libido).ti,ab.	10589

13	(dyspareunia or vaginismus).ti,ab.	5505

14	((sex or intercourse or sexual or coitus or coital) adj1 (pain or discomfort or uncomfortable or painful)).ti,ab.	1271

15	(((f?ecal or urin*) adj (dysfunction* or incontinen*)) and (intercourse or masturbat* or coitus or coital)).ti,ab.	417

16	(infertile or fertility or infertility).ti,ab.	168731

17	(("quality of life" or QOL) and (sexual* or coitus or coital or masturbat* or fertil* or infertil* or intercourse)).ti,ab.	16425

18	or/5-17	302589

19	colorectal surgery/ or surgical oncology/ or Laparoscopy/ or Robotic Surgical Procedures/ or Rectum/su	140049

20	((rectum or rectal) adj3 (surger* or operat* or procedure* or resection*)).ti,ab.	15042

21	(((laparoscopic or robot*) adj3 surger*) or laparoscopy).ti,ab.	79026

22	Total mesorectal excision*.ti,ab.	4472

23	((low anterior or abdominoperineal or surg*) adj3 resect*).ti,ab.	107376

24	or/19-23	280951

25	drug therapy/ or exp chemoradiotherapy/ or exp chemotherapy, adjuvant/ or chemotherapy, cancer, regional perfusion/ or induction chemotherapy/ or maintenance chemotherapy/	108906

26	(chemo or chemotherap* or chemoradiotherap*).ti,ab.	574485

27	exp Radiotherapy/	216405

28	(radiotherap* or brachytherap* or ((pelvis or pelvic) adj2 irradiat*)).ti,ab.	240976

29	or/25-28	882245

30	24 or 29	1125330

31	4 and 18 and 30	874

32	limit 31 to yr="1995 -Current"	797

**Embase search strategy:** Number of results before deduplication:540

Embase <1974 to 2025 Week 10>

1	exp *Rectum Cancer/	40207

2	((rectal or rectum* or anorectal) adj2 (cancer* or neoplasm* or adenocarcinoma* or lymphoma* or tumo?r* or sarcoma* or melanoma* or leiomyosarcoma*)).ti,ab.	61102

3	((colorectal adj2 (cancer* or neoplasm* or adenocarcinoma* or lymphoma* or tumo?r* or sarcoma* or melanoma* or leiomyosarcoma*)) and (rectal or rectum*)).ti,ab.	22371

4	1 or 2 or 3	78343

5	exp *Sexual Dysfunction/	44779

6	exp *Infertility/ or exp *Fertility/	104218

7	*orgasm/	1532

8	exp *incontinence/ and (exp *sexual intercourse/ or masturbation/ or orgasm/)	267

9	exp "Quality of Life"/ and (exp Infertility/ or exp Fertility/ or exp *sexual intercourse/ or masturbation/ or orgasm/)	7026

10	((sexual or erectile or ejaculat* or orgasm* or arousal) adj (dysfunction* or function* or disorder* or health or impoten*)).ti,ab.	100831

11	((sexual adj (arousal or desire)) or libido).ti,ab.	17067

12	(dyspareunia or vaginismus).ti,ab.	10537

13	((sex or intercourse or sexual or coitus or coital) adj1 (pain or discomfort or uncomfortable or painful)).ti,ab.	2283

14	(((f?ecal or urin*) adj (dysfunction* or incontinen*)) and (intercourse or masturbat* or coitus or coital)).ti,ab.	920

15	(infertile or fertility or infertility).ti,ab.	223234

16	(("quality of life" or QOL) and (sexual* or coitus or coital or masturbat* or fertil* or infertil* or intercourse)).ti,ab.	28874

17	or/5-16	388602

18	exp *rectum surgery/ or *surgical oncology/ or *cancer surgery/ or exp *laparoscopic surgery/ or exp *robot assisted surgery/ or rectum/su	188039

19	((rectum or rectal) adj3 (surger* or operat* or procedure* or resection*)).ti,ab.	23362

20	(((laparoscopic or robot*) adj3 surger*) or laparoscopy).ti,ab.	124286

21	Total mesorectal excision*.ti,ab.	7165

22	((low anterior or abdominoperineal or surg*) adj3 resect*).ti,ab.	167524

23	or/18-22	432521

24	exp *cancer adjuvant therapy/ or exp *cancer chemotherapy/	224678

25	(chemo or chemotherap* or chemoradiotherap*).ti,ab.	908572

26	exp *radiotherapy/	274378

27	(radiotherap* or brachytherap* or ((pelvis or pelvic) adj2 irradiat*)).ti,ab.	375024

28	or/24-27	1321018

29	23 or 28	1673349

30	4 and 17 and 29	1202

31	limit 30 to ("remove medline records" and yr="1995 -Current")	540

**PsycInfo search strategy:** Number of results before deduplication: 12

APA PsycInfo <1806 to March 2025 Week 2>

1	Colorectal Neoplasms/	2209

2	((rectal or rectum* or anorectal) adj2 (cancer* or neoplasm* or adenocarcinoma* or lymphoma* or tumo?r* or sarcoma* or melanoma* or leiomyosarcoma*)).ti,ab.	251

3	((colorectal adj2 (cancer* or neoplasm* or adenocarcinoma* or lymphoma* or tumo?r* or sarcoma* or melanoma* or leiomyosarcoma*)) and (rectal or rectum*)).ti,ab.	134

4	1 or 2 or 3	2430

5	exp Sexual Function Disturbances/	10295

6	exp Infertility/ or exp Fertility/	6021

7	exp orgasm/	2467

8	(Fecal incontinence/ or exp Urinary incontinence/) and ("sexual intercourse (human)"/ or same sex intercourse/ or masturbation/ or exp orgasm/)	32

9	exp "Quality of Life"/ and (exp Infertility/ or exp Fertility/ or "sexual intercourse (human)"/ or same sex intercourse/ or masturbation/ or exp orgasm/)	232

10	((sexual or erectile or ejaculat* or orgasm* or arousal) adj (dysfunction* or function* or disorder* or health or impoten*)).ti,ab.	21238

11	((sexual adj (arousal or desire)) or libido).ti,ab.	8775

12	(dyspareunia or vaginismus).ti,ab.	872

13	((sex or intercourse or sexual or coitus or coital) adj1 (pain or discomfort or uncomfortable or painful)).ti,ab.	621

14	(((f?ecal or urin*) adj (dysfunction* or incontinen*)) and (intercourse or masturbat* or coitus or coital)).ti,ab.	48

15	(infertile or fertility or infertility).ti,ab.	11313

16	(("quality of life" or QOL) and (sexual* or coitus or coital or masturbat* or fertil* or infertil* or intercourse)).ti,ab.	3888

17	or/5-16	44369

18	(exp surgery/ and colorectal neoplasms/) or Computer Assisted Surgery/	117

19	((rectum or rectal) adj3 (surger* or operat* or procedure* or resection*)).ti,ab.	54

20	(((laparoscopic or robot*) adj3 surger*) or laparoscopy).ti,ab.	347

21	Total mesorectal excision*.ti,ab.	4

22	((low anterior or abdominoperineal or surg*) adj3 resect*).ti,ab.	1346

23	or/18-22	1834

24	Chemotherapy/	3961

25	(chemo or chemotherap* or chemoradiotherap*).ti,ab.	7887

26	Radiation Therapy/	1571

27	(radiotherap* or brachytherap* or ((pelvis or pelvic) adj2 irradiat*)).ti,ab.	2213

28	or/24-27	10149

29	23 or 28	11807

30	4 and 17 and 29	32

31	limit 30 to (yr="1995 -Current" and "remove medline records")	12

**CENTRAL search strategy:** Number of results before deduplication: 138

Date Run:	14/03/2025 02:57:10

ID	Search	Hits

#1	MeSH descriptor: [Rectal Neoplasms] explode all trees	2897

#2	((rectal or rectum* or anorectal) NEAR/2 (cancer* or neoplasm* or adenocarcinoma* or lymphoma* or tumo?r* or sarcoma* or melanoma* or leiomyosarcoma*))	6011

#3	((colorectal NEAR/2 (cancer* or neoplasm* or adenocarcinoma* or lymphoma* or tumo?r* or sarcoma* or melanoma* or leiomyosarcoma*)) and (rectal or rectum*))	2919

#4	#1 OR #2 OR #3	7461

#5	MeSH descriptor: [Sexual Dysfunction, Physiological] explode all trees	3038

#6	MeSH descriptor: [Sexual Dysfunctions, Psychological] explode all trees	2852

#7	MeSH descriptor: [Infertility] explode all trees	4544

#8	MeSH descriptor: [Fertility] explode all trees	530

#9	MeSH descriptor: [Orgasm] explode all trees	165

#10	MeSH descriptor: [Urinary Incontinence] explode all trees	3552

#11	MeSH descriptor: [Fecal Incontinence] explode all trees	698

#12	#10 OR #11	4121

#13	MeSH descriptor: [Coitus] explode all trees	440

#14	MeSH descriptor: [Masturbation] explode all trees	29

#15	#9 OR #13 OR #14	604

#16	#12 AND #15	13

#17	MeSH descriptor: [Quality of Life] explode all trees	44016

#18	#17 AND (#7 OR #8 OR #9 OR #13 OR #14)	115

#19	((sexual or erectile or ejaculat* or orgasm* or arousal) NEAR/0 (dysfunction* or function* or disorder* or health or impoten*))	108

#20	((sexual NEAR/0 (arousal or desire)) or libido)	1480

#21	dyspareunia or vaginismus	1884

#22	((sex or intercourse or sexual or coitus or coital) NEAR/1 (pain or discomfort or uncomfortable or painful))	437

#23	(((f?ecal or urin*) NEAR/0 (dysfunction* or incontinen*)) and (intercourse or masturbat* or coitus or coital))	0

#24	infertile or fertil* or infertility	36330

#25	(("quality of life" or QOL) and (sexual* or coitus or coital or masturbat* or fertil* or infertil* or intercourse))	7076

#26	#5 OR #6 OR #7 OR #8 OR #9 OR #16 OR #18 OR #19 OR #20 OR #21 OR #22 OR #23 OR #24 OR #25	47035

#27	MeSH descriptor: [Colorectal Surgery] this term only	352

#28	MeSH descriptor: [Surgical Oncology] this term only	11

#29	MeSH descriptor: [Laparoscopy] this term only	7264

#30	MeSH descriptor: [Robotic Surgical Procedures] this term only	1007

#31	((rectum or rectal) NEAR/3 (surger* or operat* or procedure* or resection*))	3374

#32	(((laparoscopic or robot*) NEAR/3 surger*) or laparoscopy)	20270

#33	"Total mesorectal" NEXT excision*	763

#34	(("low anterior" or abdominoperineal or surg*) NEAR/3 resect*)	9043

#35	#27 OR #28 OR #29 OR #30 OR #31 OR #32 OR #33 OR #34	31309

#36	MeSH descriptor: [Drug Therapy] this term only	480

#37	MeSH descriptor: [Chemoradiotherapy] explode all trees	2056

#38	MeSH descriptor: [Chemotherapy, Adjuvant] explode all trees	5974

#39	MeSH descriptor: [Chemotherapy, Cancer, Regional Perfusion] this term only	149

#40	MeSH descriptor: [Induction Chemotherapy] this term only	672

#41	MeSH descriptor: [Maintenance Chemotherapy] this term only	618

#42	chemo or chemotherap* or chemoradiotherap*	111201

#43	MeSH descriptor: [Radiotherapy] explode all trees	9836

#44	(radiotherap* or brachytherap* or ((pelvis or pelvic) NEAR/2 irradiat*))	45051

#45	#36 OR #37 OR #38 OR #39 OR #40 OR #41 OR #42 OR #43 OR #44	134833

#46	#35 OR #45	160644

#47	#4 AND #26 AND #46 with Publication Year from 1995 to 2025, in Trials	138

**CINAHL search strategy:** Number of results before deduplication: 126

| **#** | **Query** | **Results** |
| --- | --- | --- |
| S80 | S44 AND S65 AND S79 | 126 |
| S79 | S73 OR S78 | 203,057 |
| S78 | S74 OR S75 OR S76 OR S77 | 166,800 |
| S77 | TI ( (radiotherap* or brachytherap* or ((pelvis or pelvic) N2 irradiat*)) ) OR AB ( (radiotherap* or brachytherap* or ((pelvis or pelvic) N2 irradiat*)) ) | 49,429 |
| S76 | (MH "Radiotherapy+") | 42,886 |
| S75 | TI ( (chemo or chemotherap* or chemoradiotherap*) ) OR AB ( (chemo or chemotherap* or chemoradiotherap*) ) | 99,735 |
| S74 | (MH "Chemotherapy, Cancer+") | 41,068 |
| S73 | S66 OR S67 OR S68 OR S69 OR S70 OR S71 OR S72 | 42,513 |
| S72 | TI ( (("low anterior" or abdominoperineal or surg*) N3 resect*) ) OR AB ( (("low anterior" or abdominoperineal or surg*) N3 resect*) ) | 16,845 |
| S71 | TI Total mesorectal excision* OR AB Total mesorectal excision* | 608 |
| S70 | TI ( (((laparoscopic or robot*) N3 surger*) or laparoscopy) ) OR AB ( (((laparoscopic or robot*) N3 surger*) or laparoscopy) ) | 14,390 |
| S69 | TI ( ((rectum or rectal) N3 (surger* or operat* or procedure* or resection*)) ) OR AB ( ((rectum or rectal) N3 (surger* or operat* or procedure* or resection*)) ) | 2,184 |
| S68 | (MH "Rectum+/SU") | 1,995 |
| S67 | (MH "Robotic Surgical Procedures") | 4,291 |
| S66 | (MH "Surgery, Laparoscopic+") | 8,466 |
| S65 | S45 OR S46 OR S47 OR S48 OR S49 OR S54 OR S57 OR S58 OR S59 OR S60 OR S61 OR S62 OR S63 OR S64 | 67,697 |
| S64 | TI ( (("quality of life" or QOL) and (sexual* or coitus or coital or masturbat* or fertil* or infertil* or intercourse)) ) OR AB ( (("quality of life" or QOL) and (sexual* or coitus or coital or masturbat* or fertil* or infertil* or intercourse)) ) | 5,487 |
| S63 | TI ( infertile or fertility or fertile or infertility ) OR AB ( infertile or fertility or fertile or infertility ) | 28,645 |
| S62 | TI ( (((f?ecal or urin*) N0 (dysfunction* or incontinen*)) and (intercourse or masturbat* or coitus or coital)) ) OR AB ( (((f?ecal or urin*) N0 (dysfunction* or incontinen*)) and (intercourse or masturbat* or coitus or coital)) ) | 78 |
| S61 | TI ( ((sex or intercourse or sexual or coitus or coital) N1 (pain or discomfort or uncomfortable or painful)) ) OR AB ( ((sex or intercourse or sexual or coitus or coital) N1 (pain or discomfort or uncomfortable or painful)) ) | 1,267 |
| S60 | TI ( dyspareunia or vaginismus ) OR AB ( dyspareunia or vaginismus ) | 1,571 |
| S59 | TI ( ((sexual N0 (arousal or desire)) or libido) ) OR AB ( ((sexual N0 (arousal or desire)) or libido) ) | 2,835 |
| S58 | TI ( ((sexual or erectile or ejaculat* or orgasm* or arousal) N0 (dysfunction* or function* or disorder* or health or impoten*)) ) OR AB ( ((sexual or erectile or ejaculat* or orgasm* or arousal) N0 (dysfunction* or function* or disorder* or health or impoten*)) ) | 21,496 |
| S57 | S55 AND S56 | 875 |
| S56 | S47 OR S48 OR S53 | 31,658 |
| S55 | (MH "Quality of Life+") | 165,126 |
| S54 | S50 AND S53 | 54 |
| S53 | S49 OR S51 OR S52 | 11,513 |
| S52 | (MH "Masturbation") | 563 |
| S51 | (MH "Sexual Intercourse+") | 10,275 |
| S50 | (MH "Incontinence+") | 14,844 |
| S49 | (MH "Orgasm") | 989 |
| S48 | (MH "Fertility+") | 7,377 |
| S47 | (MH "Infertility+") | 14,680 |
| S46 | (MH "Sexual Dysfunction, Male+") OR (MH "Sexual Dysfunction, Female+") | 11,042 |
| S45 | (MH "Sexual Dysfunction, Psychological") | 2,088 |
| S44 | S41 OR S42 OR S43 | 11,604 |
| S43 | TI ( ((colorectal N2 (cancer* or neoplasm* or adenocarcinoma* or lymphoma* or tumo?r* or sarcoma* or melanoma* or leiomyosarcoma*)) and (rectal or rectum*)) ) OR AB ( ((colorectal N2 (cancer* or neoplasm* or adenocarcinoma* or lymphoma* or tumo?r* or sarcoma* or melanoma* or leiomyosarcoma*)) and (rectal or rectum*)) ) | 1,909 |
| S42 | TI ( ((rectal or rectum* or anorectal) N2 (cancer* or neoplasm* or adenocarcinoma* or lymphoma* or tumo?r* or sarcoma* or melanoma* or leiomyosarcoma*)) ) OR AB ( ((rectal or rectum* or anorectal) N2 (cancer* or neoplasm* or adenocarcinoma* or lymphoma* or tumo?r* or sarcoma* or melanoma* or leiomyosarcoma*)) ) | 7,609 |
| S41 | (MH "Rectal Neoplasms+") | 7,439 |
| S40 | S4 AND S25 AND S39 | 126 |
| S39 | S33 OR S38 | 202,965 |
| S38 | S34 OR S35 OR S36 OR S37 | 166,704 |
| S37 | TI ( (radiotherap* or brachytherap* or ((pelvis or pelvic) adj2 irradiat*)) ) OR AB ( (radiotherap* or brachytherap* or ((pelvis or pelvic) adj2 irradiat*)) ) | 49,249 |
| S36 | (MH "Radiotherapy+") | 42,886 |
| S35 | TI ( (chemo or chemotherap* or chemoradiotherap*) ) OR AB ( (chemo or chemotherap* or chemoradiotherap*) ) | 99,735 |
| S34 | (MH "Chemotherapy, Cancer+") | 41,068 |
| S33 | S26 OR S27 OR S28 OR S29 OR S30 OR S31 OR S32 | 42,513 |
| S32 | TI ( (("low anterior" or abdominoperineal or surg*) N3 resect*) ) OR AB ( (("low anterior" or abdominoperineal or surg*) N3 resect*) ) | 16,845 |
| S31 | TI Total mesorectal excision* OR AB Total mesorectal excision* | 608 |
| S30 | TI ( (((laparoscopic or robot*) N3 surger*) or laparoscopy) ) OR AB ( (((laparoscopic or robot*) N3 surger*) or laparoscopy) ) | 14,390 |
| S29 | TI ( ((rectum or rectal) N3 (surger* or operat* or procedure* or resection*)) ) OR AB ( ((rectum or rectal) N3 (surger* or operat* or procedure* or resection*)) ) | 2,184 |
| S28 | (MH "Rectum+/SU") | 1,995 |
| S27 | (MH "Robotic Surgical Procedures") | 4,291 |
| S26 | (MH "Surgery, Laparoscopic+") | 8,466 |
| S25 | S5 OR S6 OR S7 OR S8 OR S9 OR S14 OR S17 OR S18 OR S19 OR S20 OR S21 OR S22 OR S23 OR S24 | 67,697 |
| S24 | TI ( (("quality of life" or QOL) and (sexual* or coitus or coital or masturbat* or fertil* or infertil* or intercourse)) ) OR AB ( (("quality of life" or QOL) and (sexual* or coitus or coital or masturbat* or fertil* or infertil* or intercourse)) ) | 5,487 |
| S23 | TI ( infertile or fertility or fertile or infertility ) OR AB ( infertile or fertility or fertile or infertility ) | 28,645 |
| S22 | TI ( (((f?ecal or urin*) N0 (dysfunction* or incontinen*)) and (intercourse or masturbat* or coitus or coital)) ) OR AB ( (((f?ecal or urin*) N0 (dysfunction* or incontinen*)) and (intercourse or masturbat* or coitus or coital)) ) | 78 |
| S21 | TI ( ((sex or intercourse or sexual or coitus or coital) N1 (pain or discomfort or uncomfortable or painful)) ) OR AB ( ((sex or intercourse or sexual or coitus or coital) N1 (pain or discomfort or uncomfortable or painful)) ) | 1,267 |
| S20 | TI ( dyspareunia or vaginismus ) OR AB ( dyspareunia or vaginismus ) | 1,571 |
| S19 | TI ( ((sexual N0 (arousal or desire)) or libido) ) OR AB ( ((sexual N0 (arousal or desire)) or libido) ) | 2,835 |
| S18 | TI ( ((sexual or erectile or ejaculat* or orgasm* or arousal) N0 (dysfunction* or function* or disorder* or health or impoten*)) ) OR AB ( ((sexual or erectile or ejaculat* or orgasm* or arousal) N0 (dysfunction* or function* or disorder* or health or impoten*)) ) | 21,496 |
| S17 | S15 AND S16 | 875 |
| S16 | S7 OR S8 OR S13 | 31,658 |
| S15 | (MH "Quality of Life+") | 165,126 |
| S14 | S10 AND S13 | 54 |
| S13 | S9 OR S11 OR S12 | 11,513 |
| S12 | (MH "Masturbation") | 563 |
| S11 | (MH "Sexual Intercourse+") | 10,275 |
| S10 | (MH "Incontinence+") | 14,844 |
| S9 | (MH "Orgasm") | 989 |
| S8 | (MH "Fertility+") | 7,377 |
| S7 | (MH "Infertility+") | 14,680 |
| S6 | (MH "Sexual Dysfunction, Male+") OR (MH "Sexual Dysfunction, Female+") | 11,042 |
| S5 | (MH "Sexual Dysfunction, Psychological") | 2,088 |
| S4 | S1 OR S2 OR S3 | 11,604 |
| S3 | TI ( ((colorectal N2 (cancer* or neoplasm* or adenocarcinoma* or lymphoma* or tumo?r* or sarcoma* or melanoma* or leiomyosarcoma*)) and (rectal or rectum*)) ) OR AB ( ((colorectal N2 (cancer* or neoplasm* or adenocarcinoma* or lymphoma* or tumo?r* or sarcoma* or melanoma* or leiomyosarcoma*)) and (rectal or rectum*)) ) | 1,909 |
| S2 | TI ( ((rectal or rectum* or anorectal) N2 (cancer* or neoplasm* or adenocarcinoma* or lymphoma* or tumo?r* or sarcoma* or melanoma* or leiomyosarcoma*)) ) OR AB ( ((rectal or rectum* or anorectal) N2 (cancer* or neoplasm* or adenocarcinoma* or lymphoma* or tumo?r* or sarcoma* or melanoma* or leiomyosarcoma*)) ) | 7,609 |
| S1 | (MH "Rectal Neoplasms+") | 7,439 |

**Scopus search strategy:** Number of results before deduplication: 854

(TITLE-ABS((( ( ( rectal OR rectum* OR anorectal ) W/2 ( cancer* OR neoplasm* OR adenocarcinoma* OR lymphoma* OR tumor* OR sarcoma* OR melanoma* OR leiomyosarcoma* ) ) )) OR (( ((colorectal W/2 (cancer* or neoplasm* or adenocarcinoma* or lymphoma* or tumor* or sarcoma* or melanoma* or leiomyosarcoma*)) and (rectal or rectum*)) )))

AND

TITLE-ABS(( ((sexual or erectile or ejaculat* or orgasm* or arousal) W/0 (dysfunction* or function* or disorder* or health or impoten*))) OR ( ((sexual W/0 (arousal or desire)) or libido) ) OR dyspareunia OR vaginismus OR infertile or fertility or fertile or infertility OR ( ((sex or intercourse or sexual or coitus or coital) W/1 (pain or discomfort or uncomfortable or painful)) ) OR ( (((fecal or urin*) W/0 (dysfunction* or incontinen*)) and (intercourse or masturbat* or coitus or coital)) ) OR ( (("quality of life" or QOL) and (sexual* or coitus or coital or masturbat* or fertil* or infertil* or intercourse)) ))

AND

TITLE-ABS((( ((rectum or rectal) W/3 (surger* or operat* or procedure* or resection*)) )) OR ( (((laparoscopic or robot*) W/3 surger*) or laparoscopy) ) OR "Total mesorectal excision" OR ( (("low anterior" or abdominoperineal or surg*) W/3 resect*) )) OR (( (chemo or chemotherap* or chemoradiotherap*) ) OR ( (radiotherap* or brachytherap* or ((pelvis or pelvic) W/2 irradiat*)) )))

AND

PUBYEAR > 1994 AND PUBYEAR < 2026
